# Spatial, Temporal, and Electrical Characteristics of Lightning in Reported Lightning-Initiated Wildfire Events

**DOI:** 10.3390/fire2020018

**Published:** 2019-04-03

**Authors:** Christopher J. Schultz, Nicholas J. Nauslar, J. Brent Wachter, Christopher R. Hain, Jordan R. Bell

**Affiliations:** 1 NASA George C. Marshall Space Flight Center, Huntsville, AL 35812, USA; 2 NOAA/NWS/NCEP Storm Prediction Center, Norman, OK; 3 United States Forest Service, Redding, CA; 4 Earth System Science Center, University of Alabama in Huntsville, Huntsville, AL

**Keywords:** Lightning Initiated Wildfire, Flash Density, Holdover Fires

## Abstract

Analysis was performed to determine if a lightning flash could be associated with every reported lightning-initiated wildfire that grew to at least 4 km^2^. In total, 905 lightning-initiated wildfires within CONUS between 2012 and 2015 were analyzed. Fixed and fire radius search methods showed that 81–88% of wildfires had a corresponding lightning flash within a 14 day period prior to the report date. The two methods showed that 52–60% of lightning-initiated wildfire were reported on the same day as the closest lightning flash. The fire radius method indicated the most promising spatial results, where the median distance between the closest lightning and the wildfire start location was 0.83 km, followed by a 75^th^ percentile of 1.6 km, and a 95^th^ percentile of 5.86 km. Ninety percent of the closest lightning flashes to wildfires were negative polarity. Maximum flash densities were less than 0.41 flashes km2 for the 24 hour period at the fire start location. The majority of lightning-initiated holdover events were observed in the Western CONUS, with a peak density in north-central Idaho. A twelve day holdover event from New Mexico was also discussed; outlining the opportunities and limitations of using lightning data to characterize wildfires.

## 1. Introduction

Lightning caused wildfires represent 16% of all wildfires in the last 21 years within the Continental United States (CONUS), but they are responsible for 56% of the total acreage burned [1]. Much like severe thunderstorm reports [2,3], wildfire reports can have spatial and temporal offsets, which provide challenges to developing new tools for real-time fire detection. Lightning is one way to assess these offsets due to precise spatial and temporal detection technology which pinpoints lightning occurrence. Very few studies have examined in detail spatial and temporal offsets between lightning-initiated fire reports in wildfire databases and ground-based lightning data [4–7]. The majority of wildfire reports at Kennedy Space Center were within 2 days of lightning occurrence [4]. A three day holdover criteria has been used previously to assign lightning strokes to wildfires in Australia [5]. In Northern California, 88% of reported lightning-initiated wildfires during several dry thunderstorm events had a lightning strike within 2 km of the fire start location [6].

Part of the difficulty in assigning lightning reports to specific wildfires is that fires are known to smolder for several days before rapid growth (e.g., holdover fires) [7–9]. Holdover fires are fires that smolder for days or potentially weeks before the fire is detected. The potential length of these longer duration holdover events is unknown. Using the lightning data’s spatial and temporal precision has the potential to help the community understand the land surface behavior of holdover events by pinpointing the likely time and potentially the location of the ignition source. However, an understanding of the association between lightning events and wildfire data is necessary because of the known biases in reporting other rare phenomenon like severe weather [2,3].

The occurrence of positive cloud-to-ground (+CG) lightning flashes has long been identified as being the primary initiator of lightning-initiated wildfire events because they are more likely to contain longer continuing current than their negative CG (-CG) counterparts [10–14]. A continuing current is the prolonged flow of current through the lightning channel on the order of 10s to 100s of ms, which could generate the heat required to ignite a fire [13,15–19]. It was estimated that 20% of –CG flashes contain continuing current [15]. More recent studies show that this percentage could be as high as 50% [20]. There may be too much emphasis on +CG occurrence. Recent studies also indicate that there are higher probabilities for –CG flashes to start wildfires than +CG flashes [4,6,21]. In one case, a limited sample of 6 major fire initiation days revealed that 98% of wildfires were started by -CG flashes [6].

Ground flash density is a quantity used in lightning ignition efficiency metrics used by the United States Forest Service (USFS) to assess lightning-initiated wildfire potential [22]. The flash densities used in the USFS lightning ignition efficiency product were derived from the amount of electrical current and duration of continuing current needed to ignite fuels that are commonly found in nature. Combining flash density information with 100-hr fuel moisture an empirically derived lightning metric was developed to estimate number of lightning strikes per km2 that would ignite a fire given the underlying fuel characteristics [23]. If the ignition efficiency is considered high from the 100-hr fuel moisture information, 9 flashes within a 1 km^2^ area will result in an ignition. If the efficiency is extreme, then it was postulated that 5 flashes in a 1 km^2^ area will result in an ignition [22,23]. The challenge with this empirical approach is that the performance of the ground base lightning network has changed in time, and these empirical methods may change with network performance [24,25]. It was recently shown that the average total flash (a sum of IC and CG flashes) densities across the contemporaneous United States ranged from less than 1 flash per 0.5 degree (~50 km) grid box per year in coastal regions of California to over 25 flashes per 0.5 degree grid box per year in the Southeast United States in an 18.5 year study of the NLDN data [25].

Therefore, the goal of this study is to use lightning data to understand spatial and temporal offsets between the fire report date and the time of the closest lightning flash to the fire start location. The objectives of this study are:

1. Determine the number of reported lightning-initiated wildfires with burned acreage ≥ 4 km^2^ that have a corresponding lightning flash using multiple search radii methods.
2. Understand how flash classification (in cloud (IC) or cloud-to-ground (CG)) affects this association to wildfire.
3. Understand spatial and temporal differences between lightning flash location and wildfire start location.
4. Understand flash polarity, peak amplitude, multiplicity, and flash density in the context of lightning flashes associated with lightning-initiated events.
5. Demonstrate how the lightning data can help determine the cause for wildfires with unknown or ambiguous origins.

Understanding these specific objectives can potentially help refine the wildfire database and provide an idea of the distribution of holdover events given that these fires are naturally occurring (i.e., not human ignited). Furthermore, the methods examined in this study will aid in the development of new tools that combine land and atmosphere information to determine the probability of wildfire in real-time.

## 2. Data and Methods

### 2.1. Data

#### 2.1.1. Fire Database

Reported lightning-initiated wildfires were extracted from the USFS wildfire database [26] which characterizes wildfire events from multiple federal and local agencies from 1992 to 2015. This database is regarded as the highest quality source for fire reports. The fire’s report date, report time, start date, latitude, longitude and acreage burned were the primary information utilized within this study to match with the lightning datasets. The start date and report date can differ in this database. The report date is the date in which the fire was discovered, while the start date is the date in which investigators determined the fire initiated. In this study, the report date was used as a starting point to search for the lightning because this date can be tracked back to firefighter response or satellite detection of the fire (e.g., hotspot detection, smoke).

**Figure 1:**
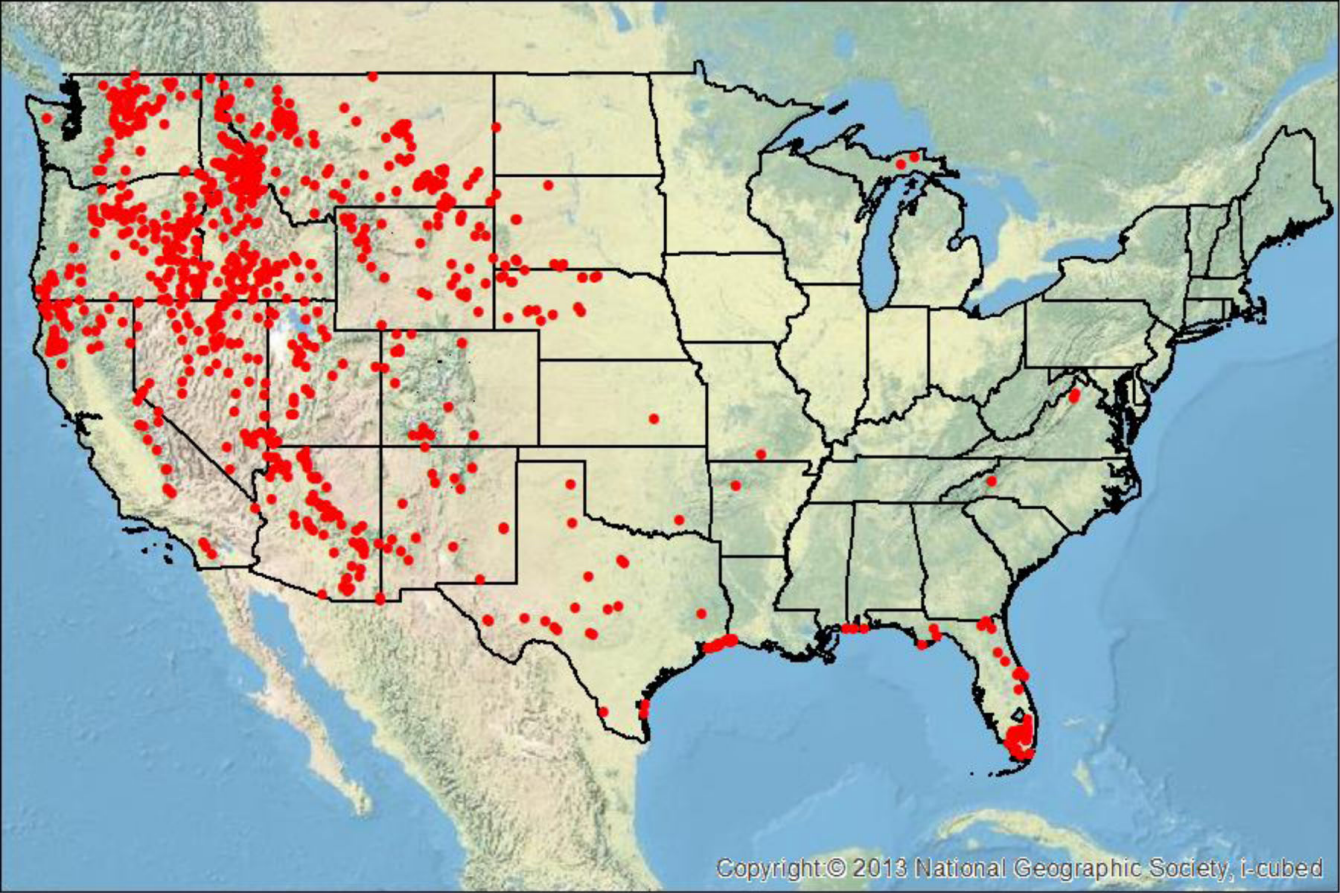
Locations of 905 lightning-initiated wildfires in the contemporaneous United States which grew to sizes ≥ 4 km^2^ between 2012 and 2015 [26]. Background topographic map from ESRI (2018).

All lightning-initiated wildfires which grew to at least 4 km^2^ between 2012 and 2015 were used for analysis. The 2012–2015 period was chosen because 2012 is the beginning of the most recent upgrade to the NLDN [24,27], and 2012 corresponds to the availability of land surface and satellite datasets (e.g., Visible Infrared Imaging Radiometer Suite (VIIRS)) currently used for wildfire detection, as well as, for the development of a real-time detection product for identification of lightning-initiated wildfires [28]. The 4 km^2^ minimum was used to examine larger fires that are on the order of the potential error in location of the NLDN system in the mountainous terrain of the Western United States [6]. Figure 1 shows the distribution of 905 wildfires that were used in this analysis between 2012 and 2015.

#### 2.1.2. National Lightning Detection Network (NLDN)

The NLDN has been used operationally for several decades and its performance is well-characterized [27,29,30]. The system consists of 113 sensors across the CONUS and has a reported flash detection efficiency of CG flashes between 90–95%, with spatial errors in CG location that are typically less than 500 m for the flash data used in this study [29]. Both IC and CG flash information were used in this study because the NLDN can misclassify flashes, especially at lower peak currents [29,31,32]. Parameters of interest from the NLDN for this study were flash time, latitude, longitude, polarity, peak current, and multiplicity.

### 2.2. Data/Output Interrogation and Statistical Analysis

A total of 1158 lightning-initiated wildfires, which consumed at least 4 km2 within the United States between 2012 and 2015. Of the 1158 fires, 253 were removed because they occurred in Alaska, where the lightning technology used in Alaska is the Time of Arrival Inc. system (TOA). This TOA system is different from the NLDN, and unlike the NLDN its accuracy metrics are not documented in the literature at the time of this study. Hawaii had zero lightning-initiated wildfires reported during this time period. Removal of the 253 Alaska fires left 905 fires within CONUS that reached an area at least 4 km^2^ (Figure 1).

Each wildfire was paired with the NLDN data in the following manner:

1. The wildfire report date and location was used to interrogate the NLDN flash data for the same date using six fixed search radii from the fire point. The fixed radii used were 10 km, 5 km, 2 km, 1 km, 500 m, and 250 m from the fire start location. These values were based on the 2 km search criteria employed in previous work [6], the median error of 500 m in the NLDN from prior to the 2003–2004 NLDN upgrade [29], the median error of 250 m cited in in most recent upgrade [27], the 90–95th observed location error of 5 km derived from observational studies [31], and an arbitrary 10 km to account for any large errors in wildfire initiation position.
2. If any lightning flash was observed within for the date and search radius, the date, time, peak current, multiplicity, distance to the fire start location and flash type (IC or CG) were recorded for each flash observed in the fixed search radius.
3. If a flash was not found, the previous day’s data were interrogated using the same search radius. This backward analysis continued until a flash was observed in the radius or the analysis went back a maximum of 14 days from the fire report date (Day 0 to Day minus 14).
4. If a flash was not observed in the 15-day period, a forward looking search was performed for one day in the event that the fire was misreported in time.
5. The start date of the wildfire report was then subtracted from the date of the closest lightning occurrence from the NLDN to determine the amount of time between the lightning occurrence and the wildfire start date within the USFS wildfire database. Any wildfire that occurred from day minus one (Day-1) to day minus 14 (Day-14) is considered a holdover event.
6. If no lightning was observed between Day+1 and Day-14, then the fire was marked as “no lightning found within 16 days and x km of fire start location.”

A secondary method was also employed that uses the fire size information to define the search criteria to assign lightning to lightning-initiated wildfire reports. This method was employed because the 2 km criteria used previously [6] may not account for potential position errors in the wildfire reporting database. Using the acreage burned information from the USFS database [26], a maximum radius of the fire was computed in km and used to search the lightning data to assign to the lightning-initiated wildfire reports. The fire start location is used as the central reference point, and then the search radius is employed in a concentric circle around the fire start location. This allows for the utilization of a fire characteristic to define the search radius, rather than a fixed radius. Because fire size varies considerably, there is larger potential error in start position in larger fires if a small fixed radius is used. A large fixed radius potentially includes lightning flashes that are not associated with the fire itself (as will be shown below). The same Steps 1–5 were followed above except that the search radius information was limited to the maximum radius of the fire and not fixed search intervals.

A Wilcoxon–Mann–Whitney rank sum test was used to determine the degree of independence between the peak current of lightning flashes that were closest to the fire start location and other flashes that occur in the fire footprint [33] (p. 159–163). +CG and –CG flash populations were tested separately. The null hypothesis used in this analysis was that the peak currents for the flashes that were “closest to fire” and “all flashes within the fire footprint” were drawn from the same distribution for each parameter examined in this study. Thus, if the p-value was p ≤ 0.05, the null hypothesis was rejected, and the peak current magnitude for a specific polarity was determined to be unique indicator of wildfire ignition.

Flash density is another commonly used metric to determine potential lightning-initiated wildfires. The CG flash density for every wildfire that contained at least one CG flash were analyzed for the 2 km fixed radius and the fire radius methods. First, a sum of the number of flashes that fell within the 2 km or fire radius was computed for each fire for the entire day that was closest in space and time to the wildfire ignition point in the USFS database [26]. Flash density was computed by taking the total number of flashes within the fixed or fire radius for 24 hours of the day in which the closest lightning flash occurred, and dividing it by the fixed area (e.g., a 2 km fixed radius would be 4π or 12.6 km^2^) or the total area burned in km^2^. This resulted in an average flash density across the entire search radius or fire footprint for the day in flashes per km^2^ per day.

## 3. Results

A total of 905 lightning-initiated wildfires that grew to at least 4 km2 between 2012 and 2015 were examined. Figure 1 shows the distribution of the 905 fires examined within the contemporaneous United States. The majority of the fires are confined to the Western United States, with an additional maximum in Southeast Texas, and Florida.

### 3.1. Distribution of Distance between Lightning Flash and Fire Start Point

Table 1 shows the percentage of wildfire events where lightning was observed within the various fixed radii and the 15 day window (Day 0 to Day-14). The 10 km search radius yielded the highest results, where 88% (797/905) of wildfire events had an IC or CG flash within the 15 day period and the 10 km search radius. The association of lightning to lightning initiated wildfires fell to 83% (757/905) if CG flashes from the NLDN were only considered. Using the 2 km search criteria [6], 60% (539/905) of wildfires could be paired with a lightning flash. The number of lightning initiated fires with at least one observed lightning flash falls to 23% (212/905) and 8% (68/905) if one were to utilize the 500 m or 250 m median location accuracy quoted for the NLDN [27,29].

**Figure 2:**
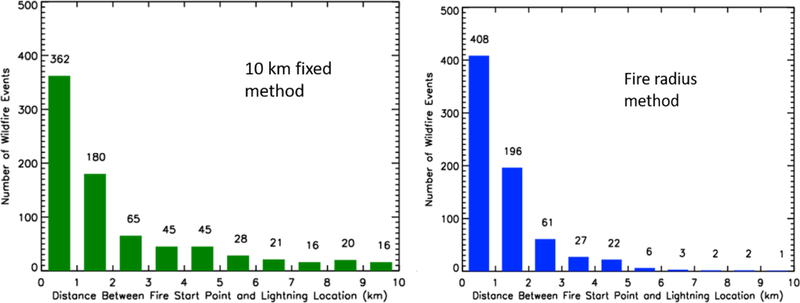
Distance between the closest lightning strike and the reported fire start location of the lightning-initiated wildfire using the 10 km fixed method (left) and the wildfire radius method (right). Distances are in 1 km increments. Numbers above each bar indicate the number of wildfire events that fell in those distance ranges for each search method.

The distance between the closest lightning flash and the wildfire start location for the 10 km search method are in Figure 2 (left). The 25^th^ percentile, median, and 75^th^ percentile distances between the closest lightning flash and wildfire start location were 0.48 km, 1.16 km, and 2.72 km for the 10 km search radius, with 101 of the wildfire-lightning distance pairs having distances greater than 5 km (Figure 2).

If the maximum radius of a fire is used as a search criteria, 81% (736/905) of wildfire events have observed lightning (IC or CG) over the fire location within 14 days of its report date (Table 1). This correspondence drops to 75% (681/905) if only lightning flashes that were identified to be a CG flash were used. The fire radius method’s median distance between the fire start location and the closest lightning flash is 0.83 km, and 408 wildfire events had lightning within 1 km (Figure 2, right). The 25^th^ and 75^th^ percentile distance between the closest flash observed by the NLDN and the fire start location were 0.39 km and 1.60 km, respectively. The 90^th^ and 95^th^ percentile values for this distance between the two points were 4.30 km and 5.86 km, and only 14 wildfire events had distances larger than 5 km (Figure 2, right).

### 3.2. Distribution of Fires in Time

**Figure 3:**
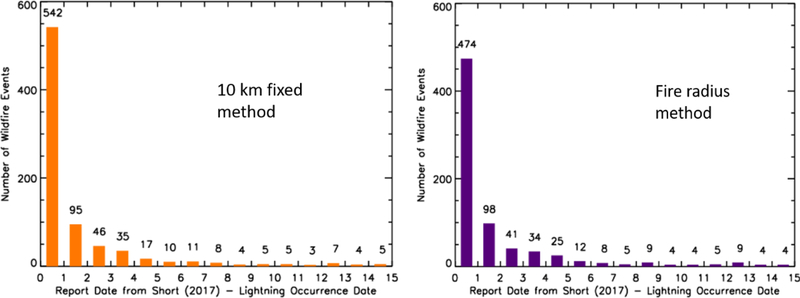
Difference in days between the wildfire report date in the USFS database [26] minus the date of the closest lightning flash for the fixed 10 km method (left) and the fire radius method (right).

The focus of this section is to understand the temporal correspondence between lightning occurrence and fire report time. Using the fixed 10 km spatial criteria, the largest frequency was on Day 0 with 60% (542/905) of fires reported the same day as lightning occurrence (IC and CG). This number of fires increased to 75% (683/905; Figure 3, left; Table 2) if a three day holdover window was used, and 84% (756/905) of lightning-initiated wildfires were accounted for in a seven day holdover period. For the 2 km radius, the Day 0, three day holdover, and seven day holdover percentages were larger but trended in a similar manner (44%, 56%, and 59%, respectively).

Utilizing the maximum wildfire radius search methodology, the largest percentage of fires were once again reported on the same day as the lightning (52%; 474/905). Just over 71% (643/905) of fires were observed within the three day holdover period. This increases to 77% (697/905) if a seven day holdover window is used (Table 2, Figure 3, right).

Interestingly, a number of wildfires had the closest lightning the day after the report date within the wildfire database (Table 2). This means that this small number of wildfires are possibly misreported in the database, an existing fire created lightning in a pyrocumulus cloud that was associated with the fire using the present methods [34,35], or the NLDN potentially missed a low amplitude CG flash [29,31,32]. The fixed radius methods showed that up to 7% of the number of wildfires had the closest lightning the day after the wildfire report. Only ~1% of wildfires had the closest lightning the day after the event.

**Figure 4:**
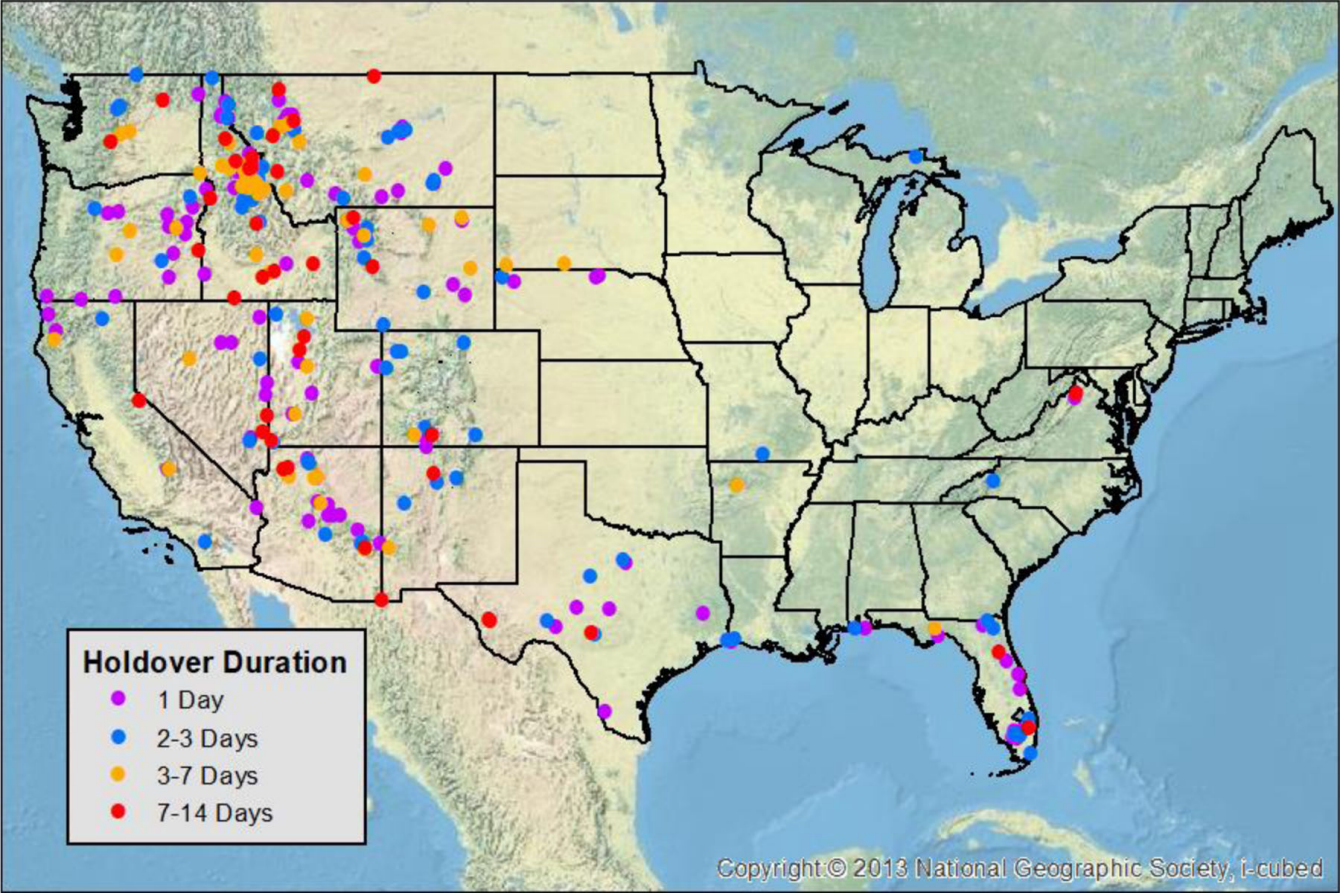
Locations of lightning-initiated holdover wildfires in the contemporaneous United States which grew to sizes ≥ 4 km^2^ between 2012 and 2015. Background topographic map from ESRI (2018).

Spatially, the majority of holdover events were located in the western United States (Figure 4). Every USFS Geographic Area Coordination Center, and half of the states within CONUS had at least one holdover event between 2012 and 2015. The highest concentration of holdover events was in north-central Idaho, with other areas like the Florida Everglades, central Arizona, eastern Oregon, and northwest Wyoming having larger concentrations of events.

### 3.3. Flash Polarity, Peak Amplitude, and Multiplicity

**Figure 5:**
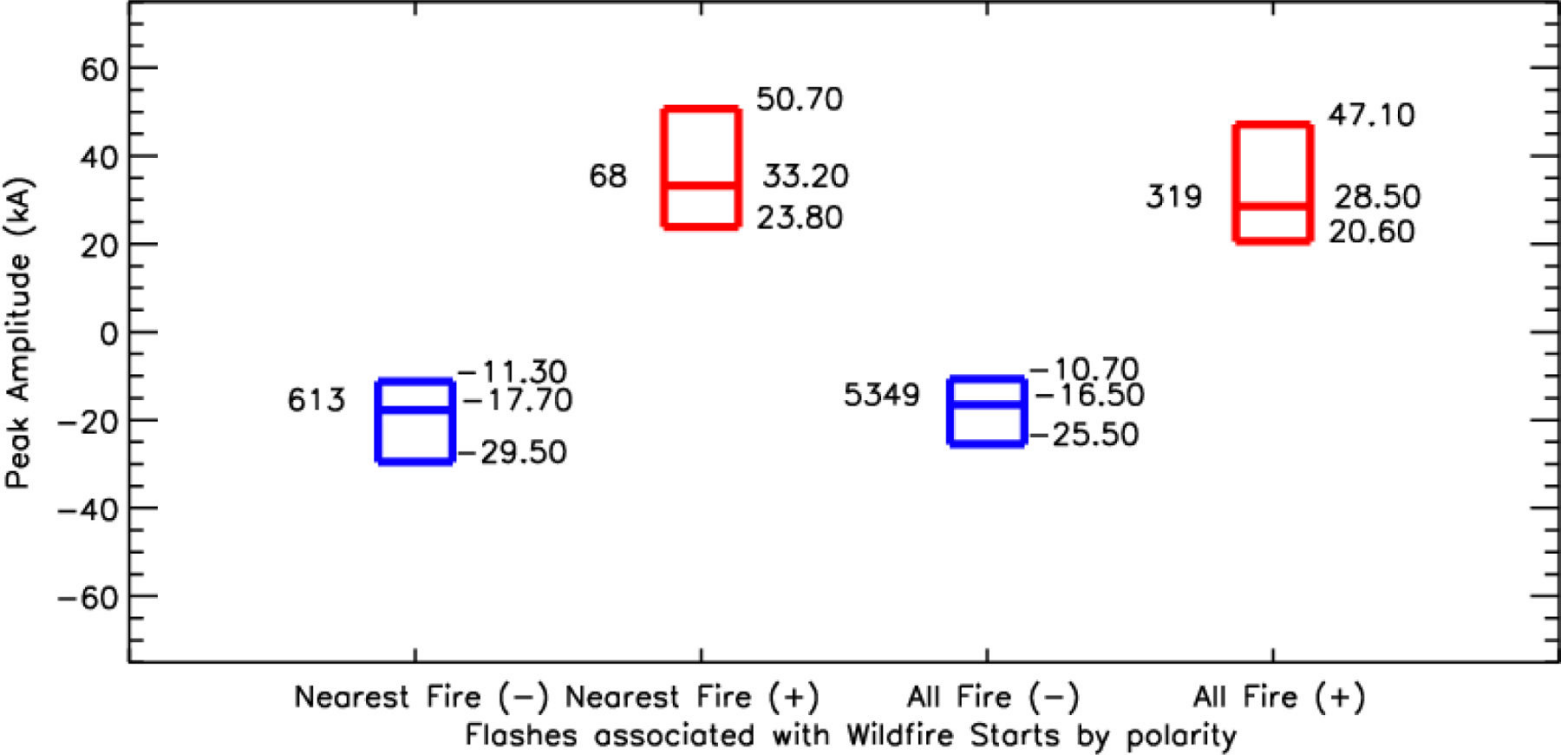
Distribution of polarity and peak amplitude for the closest flash to the fire start location, and all flashes that fall within the fire radius on the day of lightning occurrence. Blue boxes represent negative polarity, red boxes positive polarity, the number to the left of each box indicates the number of flashes, and the numbers on the right of each box are the 25^th^, median, and 75^th^ percentile peak amplitudes observed.

Previous work emphasizes the role of polarity in wildfire starts. To determine the role of polarity, peak amplitude, and multiplicity in lightning-initiated wildfire starts, the 681 cases where a CG flash was observed in the wildfire’s radius were used. The CG flash that was closest in space and time to the wildfire start location were analyzed (Figure 5). The results show that 90% (613/681) of lightning flashes that were closest to the fire start location were of negative polarity. This percentage is in line with recent climatology work for the NLDN in CONUS [25], which showed that the percentage of +CG flashes ranged from 4 to 20%. The median peak amplitude of these flashes were −17.7 kA, with a 25^th^ percentile of −11.3 kA, and a 75^th^ percentile of −29.5 kA. Forty-six percent (286/613) of negative CG flashes had a multiplicity of one, with a maximum multiplicity of 12. The 68 positive lightning flashes that were closest to the wildfire start location were observed to have characteristics similar to that observed in previous studies [13,31,32,36]. The median, 25^th^, and 75^th^ percentile peak amplitudes were 23.8 kA, 33.2 kA, and 50.7 kA. Eighty percent (55/68) flashes had a multiplicity of 1, and the maximum multiplicity observed was 7 strokes.

Next, additional CG flashes that fell within the fire footprint were examined to directly compare with the flash that was designated as the firestarter. A total of 319 +CG and 5349 –CG flashes occurred within the footprint of the 681 fires that contained at least 1 CG flash on the same day as the closest CG flash in space and time. A first glance at Figure 5 indicates that the median, 25^th^, and 75^th^ percentile peak amplitudes are very similar. Wilcoxon-Mann-Whitney statistical testing indicated that the “closest to fire” and “all flashes within footprint” populations were inconclusive to determine independence when looking at each polarity independently. For –CG flashes, the Z-score was 2.794 with a p-value of 0.0026 when directly comparing the 613 –CG flashes closest to the fire to the remaining 5349 –CG flashes that fell within fire footprints. Given the disparity in sample size (e.g., 5349 –CG in the vicinity of the fire to the 613 –CGs closest to the fire start), 10 random populations of –CG flashes were chosen from the “within fire footprint” population that were on the order of the same size as the “closest to fire” population to statistically compare similar sample sizes. Only 4 of the 10 comparisons between randomly selected populations of –CG flashes that occurred within a fire footprint and the closest -CG had Z-Scores ≥ 1.96 and p-values less than 0.025.

Statistical independence was not observed for the +CG flash populations. The Z-score and p-value for the +CG flashes were 1.60 and 0.05, respectively. Ten random samples were also chosen from the population of 319 +CG flashes that were closest to the fire. Only one of the ten comparisons between the randomly sampled +CG population and the closest to wildfire +CG flash population had a Z-score ≥ 1.96. Thus, there does not appear to be something significantly different that would allow one to separate a firestarter from a non-firestarter based on peak amplitude alone. However, when combined with additional meteorological or land surface information, the CG location information will be vital to pinpointing the location of fire starts.

### 3.4. Flash Density

Flash density has been used as an important metric for forecasting lightning-initiated wildfire events. Given the dependence on flash density for forecasting applications and ignition efficiency in the USFS, this study briefly examined the flash densities observed at the wildfire location. Using the 2 km fixed radius, the 25^th^, median, and 75^th^ percentile flash densities were 0.07 flashes km^−2^ (1 flash per 12.6 km^2^). Expanding this to a 5 km fixed search methods, the 25^th^, median, and 75^th^ percentile total flash counts were 2, 4, and 9 flashes. This resulted in flash densities of 0.02, 0.05, and 0.11 flashes km^−2^ for the 24 hour period in which the closest lightning flash to the fire start location was observed. The wildfire radius search radius 25^th^, median, and 75^th^ percentile total flash counts were 2, 4, and 10 flashes. Normalizing these numbers by fixed search area or wildfire size makes the flash densities very small as well, with the 25^th^, median, and 75^th^ percentile flash densities of 0.08, 0.19, and 0.41 flashes km^−2^ for a 24 hour period. Thus, the flash densities utilized for forecasting purposes and lightning ignition efficiency metrics are likely too high when compared to observed flash densities in the vicinity of wildfire starting locations.

## 4. Discussion

### 4.1. Reasons for Lack of Lightning for Reported Lightning-Initiated Events

There are three main reasons why a reported lightning-initiated event could not be paired with a lightning flash. The first reason could simply be that the fire report was misclassified as lightning-ignited when the cause was human related. Literature shows that 84% of wildfires are started by human activities [1]. Therefore, some of these events may be human caused, but because there were thunderstorms somewhere near the fire start location, they were reported as lightning-initiated. However, based on the experience of the operational fire community and the authors of this paper, misreporting is the least likely reason given the extensive methods used for fire investigation.

**Figure 6:**
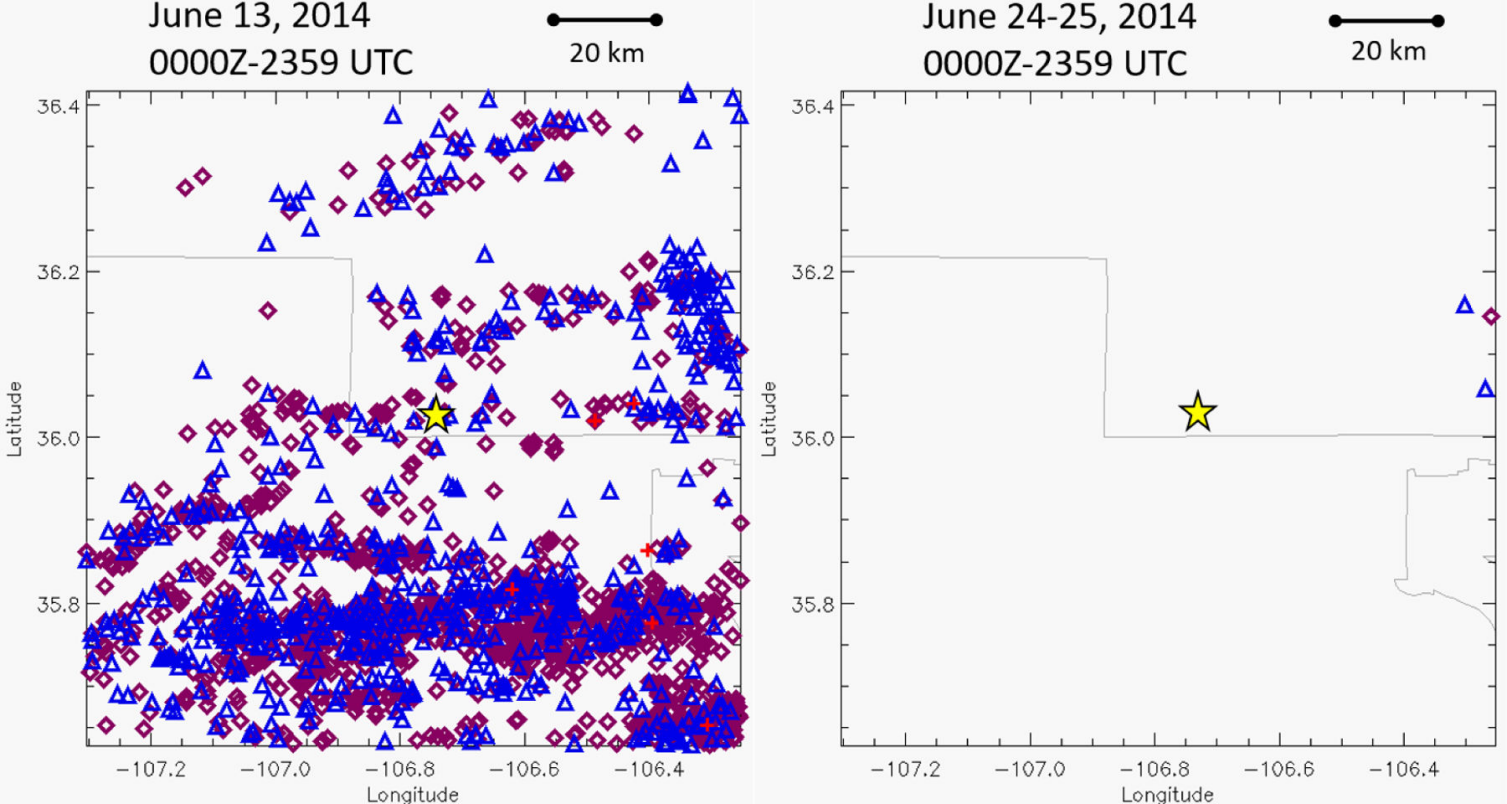
Lightning strikes near the Diego Fire in north-central New Mexico on 13 June 2014 and 24–25 June 2014. Purple diamonds are IC flash locations, blue triangles are –CG flash locations, and red plus signs are +CG flash locations according to the NLDN. The fire start location is represented by the yellow star at latitude 36.0767, longitude −106.7300.

The second reason why there may not be lightning within the 15 day period used in this analysis around the fire report date is that the community does not have an accurate grasp on how long lightning-initiated holdover fires can smolder [7]. It is known that soil and vegetation type can help a fire smolder for long periods of time, especially in the boreal forests, where peat can allow a fire to burn for several months below snow and ice [37]. The majority of these events were observed in the western United States, with the largest density observed in north-central Idaho (Figure 6). Holdover events beyond seven days can occasionally be a challenge to proper separating lightning from human activity. For example, there were three 7+ day events that occurred in densely populated areas of Florida and northern Virginia.

The third reason is that there is the potential that the lightning flash was not detected, especially if the peak amplitude was weak. A summary of several field campaigns that show the NLDN’s detection efficiency for CG flashes was on the order of 90–95% [29,31,32]. The minimum detectable peak current from these flashes was 4–6 kA, and this threshold varied with space throughout the CONUS. It is not fully clear how recent upgrades to the algorithms have affected the performance of these data since the time of those field studies [29,31,32]. However, recent analysis suggests that that the CG detection of the NLDN should be similar if not better [30]. Importantly, the most variation in detection occurs in the western CONUS where the majority of the lightning initiated starts are located in this study (e.g., Figure 1), and is supported by analysis for both the NLDN [29,38] and Earth Networks Total Lightning Network (ENTLN) [39–41].

### 4.2. Holdover Fires

Holdover wildfires present a challenge to a real-time lightning-initiated wildfire detection methodology. Methodologies for real-time prediction [28] need accurately collocated lightning and wildfire reports to understand the underlying surface and meteorological conditions at the time of lightning occurrence. There is not a reliable database for understanding the duration of holdover fires [7]. Literature varies on these dates between two and seven days [4,5,8]. The two week period before the reported fire date was a first guess to determine how far back one could go to confidently assign lightning data to a particular lightning-initiated wildfire.

In the present study, a fire was considered a holdover event if the wildfire start date within the USFS wildfire database was different than the date of the closest flash to the fire start location. Positively, 50% of the lightning-initiated wildfires in the USFS wildfire database are reported within the same day as the closest lightning flash to the fire start location observed by the NLDN. Another 23% of wildfires are reported according to the USFS wildfire database within 5 days of the closest flash occurrence. Therefore, there should be a suitable population of lightning-initiated flashes to understand the dichotomy between fires that are immediately detected or observed and those that smolder for days within other datasets important to wildfire characterization (e.g., live and dead fuel moisture, soil moisture, rainfall, local relative humidity, wind, etc.)

The longest duration holdover event analyzed in this sample was twelve days. According to the USFS wildfire database [26], a lightning-initiated wildfire was reported on 29 June 2014 in north-central New Mexico. The start date that was provided to the USFS wildfire database was 25 June 2014, indicating that several days passed between the identification of the fire and when it was thought to have started. However, in this analysis, no lightning strike was located within 10 km of the fire start location on 25 June and no thunderstorms occur between the 25 June start date in the USFS wildfire database and the report date of 29 June 2014 (Figure 6). Looking backward from the 25 June 2014 start date reported, the only lightning within wildfire’s perimeter is on 13 June 2014 (Figure 6). Examining the lightning events within that 3.8 km shows the closest lightning flash is within 77 m of the fire start location, which is well below the median error of 250 m or 500 m [27,29]. This is likely an extreme case for a holdover fire in north central New Mexico, as most fires are within the 0–7 day period (Figures 1 and 4). Fire misclassification was also possible because the fire start location was near a motorized vehicle trail. The authors also realize that with holdover events, formal definition of a wildfire start date differs much like the challenges of formally defining the start of a tornado in time [42]. Therefore, with increasing holdover duration, additional scrutiny must be used to understand all possible factors before the fire is utilized in any wildfire prediction algorithms.

### 4.3. Fixed vs Fire Radius Search Methods

One of the key observations was differences between the number of lightning-wildfire pairs for the 10 km and fire radius search methods (88% vs 81%; Table 1). Digging deeper and looking at the distributions of the distances between the closest lightning flash to the wildfire start location, the fire radius method produces more pairs that are closer to the fire start location. In Figure 2 the fire radius search method had 408 lightning-initiated fires where the closest flash is within 1 km as compared to the 362 lightning-initiated fires at 10 km (Figure 2). Within 5 km of the fire start location, the fire radius method continues to have more lightning-initiated fires with lightning flashes closer to the fire start location than the 10 km fixed radius (714 vs 697). The 5 km range is important because it is approximately between the 90^th^ and 95^th^ percentile distance error for the NLDN [31].

Differences between the two search criteria were also prevalent beyond 5 km. A total of 101 lightning-initiated fires had the closest lightning over 5 km away from the fire start location using the 10 km fixed radius, as compared to only 14 lightning-initiated fires with the closest lightning at distances greater than 5 km using the fire radius search method. While all 14 of the fires using the fire radius search method had radii greater than 5 km, only 38 of the fires that were paired with lightning occurrence in the 10 km fixed radius had grown to sizes where the fire radius was larger than 5 km. This means that 63 of the 101 fires-lightning pairs using the 10 km search method had distances between the fire start point and the closest lightning flash that were larger than the radius of the lightning-initiated wildfire. Those 63 fire-lightning pairs account for 7% (63/905) of the overall population of fires in this analysis, which is approximately the difference between the 10 km fixed search and the fire radius method (Table 1; 88% vs 81%). Furthermore, all 63 fire-lightning pairs occur on Day 0, explaining most of the difference between the 10 km fixed method and the fire radius method in Figure 3 in the Day 0 category (542 vs 474). This information infers that there were thunderstorms in the area on the same day as ignition for the additional 63 lightning-fire pairs captured in the 10 km search method, it was assumed that the thunderstorms in the area of the fire location were responsible for the fire. It is unclear why the observed NLDN lightning for these cases falls outside each wildfire’s radius used in the radius search.

While the 10 km fixed method was able to assign lightning to more reported lightning-initiated wildfires in the USFS wildfire database in this study period, it appears that the fire radius method is able to assign lightning that was closer in space and time to the wildfire start location. This information is impactful because it would increase confidence that the correct set of flash dates, times, and locations were identified, which helps in the diagnosis of critical fire weather variables at the time of ignition.

However, fire size is not always available. Therefore, if a fixed boundary is desired because of an unknown fire size, it is suggested that the boundary not exceed 5–6 km to confidently assign lightning to a wildfire based on this analysis. This range fits within the observed 90^th^ and 95^th^ (4.30 km and 5.86 km) percentiles for distance between fire start and lightning locations and around the maximum location error for the NLDN [31]. It was shown that 77% of wildfires had a lightning flash within 5 km of the wildfire start location.

### 4.4 The use of NLDN flash level data versus stroke level data

In the present study, NLDN flash data were preferred over the NLDN stroke data because of the ability to assess flash multiplicity and compare with previous work [e.g., 10–12]. However, another dataset that is available to the community is NLDN stroke level data, which are the locations of individual ground connections that make up a flash. The stroke-level data are clustered to produce the NLDN flash dataset using a spatial criteria of 10 km and a maximum temporal criteria of 1s [43]. Temporal intervals between strokes cannot exceed 500 ms, and the maximum number of strokes per flash is 15 [43]. Multiple NLDN strokes can make up an individual NLDN flash, and the location, timing, and peak amplitude of the first stroke is recorded in the NLDN flash dataset. The number of strokes per flash is reported as multiplicity in the flash dataset.

The same set of lightning-initiated wildfires was examined using the stroke level data and the fire radius method. The reanalysis indicates that the same number of fires were identified, with very similar spatial offsets from the fire start location. Therefore, NLDN stroke level data can be used for analysis, which is an important consideration for any future real-time lightning initiation wildfire product given some operational users of lightning data only receive the stroke level data.

## 5. Conclusions

Reported lightning-initiated wildfires that grew to at least 4 km2 from 2012–2015 were examined to determine the number of events that could be associated with a lightning flash. In total 1158 events occurred within the United States during this 4 year period, with 905 of them residing within CONUS. In total a 15 day search criteria from the report date was used to assign lightning flashes to reported lightning-initiated wildfire events. Key observations were as follows:

1. Using the 2 km search radius [6], 60% of wildfires had at least one lightning flash within 2 km of the wildfire start location. The 10 km search radius yielded the highest number of reported lightning-initiated wildfires that contained a least one observed lightning flash. A total of 88% (797/905) of wildfire events had an IC or CG flash within the 15 day period and the 10 km search radius. However, the mean and 25^th^/75^th^ percentile distances between the fire start location and the closest lightning flash in space were the largest of the search criteria, and 63 of the lightning-wildfire pairs fell outside of the fire’s boundaries.
2. Using the fire’s radius as a search criteria, 81% of reported lightning-initiated events could be matched within 15 days of lightning-initiated wildfire report date. The median distance between the closest lightning flash and the fire start location was 0.83 km. The 75^th^ percentile was 1.6 km, and the 95^th^ percentile distance was 5.86 km.
3. The largest percentage of lightning-initiated wildfires were reported on the same day as the lightning event. A total of 71% and 77% of wildfire events are reported within three days and seven of a lightning flash, respectively. Approximately ~1% of reported lightning initiated wildfires had the closest lightning flash the day after the reported ignition time.
4. Negative CG flashes accounted for 90% (613/681) of the closest lightning flashes to fire start locations. Forty-six percent of –CG flashes had a multiplicity of one. Positive CG flashes only accounted for 10% (68/681) of the CG flash population that was closest to the fire start location. Eighty percent (55/68) of +CG flashes had a multiplicity of 1. Peak amplitude was not observed to be statistically different between flashes closest to the fire, and other lightning flashes within the fire footprint.
5. Flash densities were observed to be smaller than many of the metrics used for lightning-initiated wildfire forecasting (e.g., lightning activity level, lightning ignition efficiency). The majority of flash densities were less than 0.41 flashes km^−2^, which is far less than current lightning ignition efficiency metrics of several flashes per km^2^.

Based on the analysis above, the fire radius method is more ideal than a fixed method to locate the time and location of lightning-initiated fire starts because it includes a physical characteristic of the fire, which adjusts the radius’ size. The addition of the spatial and temporal information in the NLDN data provides extra confidence in the start location, which is key to reconstruct land surface, precipitation, and near surface weather conditions at the time of ignition. If a fixed radius is needed due to unknown fire size, a 5 km search radius provided similar results to the fire radius method. Additional detailed analysis that is beyond the scope of this paper is needed to characterize the wildfire footprint and NLDN locations for the 63 lightning-wildfire pairs which had the lightning outside of the fire’s footprint.

Future work should continue to investigate the number of wildfires that were reported as lightning-initiated, but no lightning was observed using the criteria above. This would help to determine the maximum duration of a holdover wildfire, help further characterize the variability within the fire database, and identify the frequency of classification errors within the fire occurrence database. Ultimately, characterizing holdover wildfires will allow us to understand how the land surface changes with time after the initial lightning flash that causes the ignition. The ultimate goal of the identification of lightning-initiated fires in real-time is to provide more information for decision makers to have a positive impact on fire management and response. This work helps to understand conditions in the past to help predict lightning-initiated wildfires in the future.

## Figures and Tables

**Table 1.** Percentage of lightning events associated with reported lightning-initiated wildfires in space using fixed search criteria and the fire radius criteria from Day 0 to Day-14.

	10 km	5 km	2 km	1 km	0.5 km	0.25 km	Fire Radius
All Flashes	88.07%	76.69%	59.56%	39.67%	23.43%	7.51%	81.33%
CG Only	83.65%	71.60%	53.81%	33.59%	18.90%	6.30%	75.25%

**Table 2.** Percentage distribution of lightning events with time using fixed search criteria and the fire radius method.

Day	10 km	5 km	2 km	1 km	0.5 km	0.25 km	Fire rad (IC+CG)	Fire Rad (CG only)
−14	0.55%	0.22%	0.00%	0.00%	0.00%	0.00%	0.44%	0.33%
−13	0.44%	0.33%	0.00%	0.00%	0.00%	0.00%	0.44%	0.22%
−12	0.77%	0.44%	0.22%	0.22%	0.11%	0.11%	0.99%	0.88%
−11	0.33%	0.11%	0.11%	0.00%	0.00%	0.00%	0.55%	0.55%
−10	0.55%	0.11%	0.00%	0.00%	0.00%	0.00%	0.44%	0.44%
−9	0.55%	0.22%	0.00%	0.00%	0.00%	0.00%	0.44%	0.44%
−8	0.44%	0.44%	0.33%	0.22%	0.22%	0.00%	0.99%	0.66%
−7	0.88%	0.33%	0.33%	0.11%	0.00%	0.00%	0.55%	0.44%
−6	1.22%	0.99%	0.33%	0.11%	0.00%	0.00%	0.88%	0.22%
−5	1.10%	0.88%	0.66%	0.11%	0.00%	0.00%	1.33%	0.88%
−4	1.88%	1.66%	1.44%	0.99%	0.77%	0.11%	2.76%	2.54%
−3	3.87%	2.98%	2.21%	1.77%	0.88%	0.33%	3.76%	3.43%
−2	5.08%	4.09%	2.76%	2.21%	1.10%	0.55%	4.53%	3.87%
−1	10.50%	9.39%	7.51%	4.97%	3.31%	0.22%	10.83%	10.17%
0	59.89%	54.48%	43.65%	28.95%	17.02%	6.19%	52.38%	50.17%
1	3.76%	7.29%	7.07%	5.41%	3.20%	1.44%	1.10%	1.10%
